# Efficient conversion of distillers grains as feed ingredient by synergy of probiotics and enzymes

**DOI:** 10.3389/fmicb.2024.1403011

**Published:** 2024-07-04

**Authors:** Kai Chen, Xiangrong Deng, Dahai Jiang, Lanxian Qin, Mengqi Lu, Wenxuan Jiang, Manqi Yang, Liangliang Zhang, Jianchun Jiang, Liming Lu

**Affiliations:** ^1^Academy of Advanced Carbon Conversion Technology, Huaqiao University, Xiamen, China; ^2^Fujian Provincial Key Laboratory of Biomass Low-Carbon Conversion, Huaqiao University, Xiamen, China; ^3^College of Chemical Engineering, Huaqiao University, Xiamen, China; ^4^Institute of Chemical Industry of Forest Products, Nanjing, China

**Keywords:** distillers grains, fermented feed, probiotics, enzyme, anti-nutritional factors

## Abstract

The direct feeding value of distillers grains is low due to the presence of higher cellulose, lignin and anti-nutritional factors such as mannan and xylan. In this study, complex enzymes and probiotic flora based on “probiotic enzyme synergy” technology were used to produce fermented distillers grains. The optimal substrate ratio, moisture content, fermentation time and temperature were determined. Subsequently, scale-up experiments were conducted to determine the performance of fermented feed. The results showed that multi-probiotic (*Lactobacillus casei*, *Bacillus subtilis*, *Saccharomyces cerevisiae*, and *Aspergillus oryzae*) cooperated with complex enzymes (glucanase, mannanase, xylanase) showed excellent fermentation effect, crude protein, trichloroacetic acid soluble protein and fat increased by 31.25, 36.68, and 49.11% respectively, while crude fiber, acidic fiber and neutral fiber decreased by 34.24, 26.91, and 33.20%, respectively. The anti-nutritional factors mannan and arabinoxylan were reduced by 26.96 and 40.87%, respectively. Lactic acid, acetic acid, and propionic acid in the fermented organic acids increased by 240.93, 76.77, and 89.47%, respectively. Butyric acid increased significantly from scratch, and the mycotoxin degradation effect was not significant. This study provides a potential approach for high-value utilization of distillers grains.

## 1 Introduction

The shortage of feed raw materials, especially protein feed, is a huge problem facing the Chinese feed industry (Xiong and Yang, 2021). Liquor grains refer to the by-product produced by microbial fermentation in the industrial production of liquor. Its composition varied according to the crop raw materials such as corn, wheat and sorghum used in brewing and the process conditions used in the brewing process. But in general, distillers grains usually contain a lot of organic matter and have a high nutritional level. It is rich in crude protein and crude fiber, accounting for about 13–27% and 16–28%, respectively. Followed by less ash and fat, accounting for 9–13% and 3–10%, respectively, (Liu, 2011). In 2022, the amount of liquor grain production in China had reached more than 20 million tons. The huge production of distillers grains has laid a good foundation for the development of the distillers grain resource utilization industry. Although distillers grains have high nutritional value, its high dietary fiber content limits its application in pig breeding. Directly applying distillers grains to pig feed will lead to low digestion efficiency and even cause stress in animals due to anti-nutritional factors in the raw materials, such as mannan, arabinoxylan, and glucan (Kim and Duarte, 2021). Therefore, we need to find an effective method to improve the performance of distillers grains efficiently and enhance its application value in the feed industry.

Many studies have shown that multi-enzymes can have effective effects on feed utilization of non-starch polysaccharides (NSPs), including improving growth and animal health, due to synergistic effects by enzymes. For instance, adding complex multi-enzymes (protease, α-amylase, β-glucanase and xylanase) to the feed of weaned piglets improves the utilization rate and the diversity of hindgut fecal microorganisms (Park et al., 2020). There are also many studies showing that the use of microorganisms for solid-state fermentation can transform miscellaneous meal, optimize its nutritional composition, and improve its feeding value in pig production efficiently. Czech added 8% fermented rapeseed meal to piglet diets, which had a beneficial effect on the immune status of piglets (Czech et al., 2021); *Bacillus subtilis* and *Enterococcus faecalis* were used to conduct two-stage fermentation to improve the digestibility of dry matter and crude protein of corn-soybean meal mixed feed (Shi et al., 2017). The research found that fermented cotton meal feed could improve the feed conversion rate and intestinal barrier function of weaned piglets by regulating intestinal flora (Gu et al., 2021).

Adding probiotics to the diet, in addition to anti-toxin and reduce diarrhea, can also improve the intestinal health and nutrient digestibility of pigs, thereby benefiting the pigs’ nutrient utilization and growth performance (Liu et al., 2018). *Aspergillus oryzae*, *Saccharomyces cerevisiae*, *Lactobacillus casei*, and *Bacillus subtilis* are probiotic strains that are allowed to be added to feed according to the “Feed Additive Species Catalog” promulgated by the Ministry of Agriculture and Rural Affairs of China, and can be used to develop a variety of unconventional feed ingredients (Nunes et al., 2018). *Aspergillus oryzae* and *Bacillus subtilis* are aerobic microorganisms, *Saccharomyces cerevisiae* is a facultative anaerobic microorganism, and *Lactobacillus casei* is an anaerobic probiotic. The composite flora could ensure that probiotics were growing and metabolizing vigorously at all stages of fermentation, which improved the performance of feed ingredient fermentation (Ilango and Antony, 2021). However, there are currently few studies on the use of multiple probiotics to ferment white distillers grains with complex enzymes, and the research on the nutritional value of distillers grains co-fermented by probiotics and enzymes is still unclear. Therefore, this article aims to study the effects of nutrients, anti-nutritional factors, mycotoxins using *Aspergillus oryzae*, *Saccharomyces cerevisiae*, *Lactobacillus casei*, *Bacillus subtilis* and complex enzyme reagents to ferment distillers grains, then establish optimal fermentation conditions, in order to open up new areas for developing the pioneering exploration of new stable and functional fermented biological feeds.

## 2 Materials and methods

### 2.1 Instruments and reagents

#### 2.1.1 Substrate for fermentation

The fermentation substrates: distillers grains, bran, soybean meal, corn and molasses used in this study were all sourced from the local market and stored at room temperature (about 25°C) in a cool and dark cabinet. The nutritional composition of the fermentation substrate is shown in Table 1.

**Table 1:** Nutrient composition of fermentation substrates.

Sample	Crude protein (CP), %	Crude fiber (CF), %	Fat, %
Distillers grains	19.70	3.42	6.10
Corn	12.10	7.36	7.30
Wheat bran	18.50	3.81	8.20
Soybean meal	37.60	0.94	12.90
Molasses	9.70	0.25	0.80

#### 2.1.2 Probiotics

The probiotics used in this study were *Aspergillus oryzae* (CGMCC No. 18109), *S. cerevisiae* (CGMCC No. 28099), *Lactobacillus casei* (CGMCC No. 8149) and *Bacillus subtilis* (CGMCC No. 8148) were from the laboratory’s original collection and stored in China General Microbiological Culture Collection Center (CGMCC). *Aspergillus oryzae* was cultured in modified Marsden medium at 30°C and 200 rpm for 48 h, *Saccharomyces cerevisiae* was cultured in YPD medium at 30°C and 200 rpm for 24 h, *Lactobacillus casei* was cultured in MRS medium at 37°C and 200 rpm for 12 h, and *Bacillus subtilis* was cultured in LB medium at 37°C and 200 rpm for 12 h.

#### 2.1.3 Complex enzyme

The enzyme preparation used in this study is composed of multiple enzymes, including xylanase activity ≥20,000 U/g; β-glucanase activity ≥2,500 U/g; β-mannanase activity ≥600 U/g; fiber enzyme activity ≥800 U/g; amylase activity ≥3,000 U/g.

#### 2.1.4 Instruments and equipment

Kjeldahl nitrogen determination instrument K1100F (Haineng, Shandong, China).

Fiber Digestion Apparatus F2000 (Haineng, Shandong, China).

Fat analyzer SOX606 (Haineng, Shandong, China).

High Performance Liquid Chromatograph 1,260 Infinity II (Agilent, United States).

### 2.2 Optimization of fermentation substrate ratio

In order to obtain excellent fermentation results, the following experiments were designed to explore the optimal fermentation moisture content and appropriate ratio of the fermentation substrate. The fermentation effect was evaluated based on the crude protein content and acid-soluble protein content after fermentation. The probiotic liquid of *Aspergillus oryzae*: *Saccharomyces cerevisiae*: *Lactobacillus casei*: *Bacillus subtilis* is added in a ratio of 1:1:1:1. The total amount added is 10% of the substrate. The amount of compound enzyme preparation is 1‰ of the substrate. The mixture was stirred evenly, put into one-way valve fermentation bags, and fermented at room temperature (28–33°C) for 5 days.

#### 2.2.1 Suitable moisture content for fermentation

Traditional fermented feed was used 20% corn, 20% soybean meal and 60% wheat bran as substrates, the fermentation effects of the substrates at moisture contents of 10, 30, and 50% were explored.

#### 2.2.2 Fermentation substrate

The 90% bran group was took as a control, the fermentation effect of the substrate under different distillers grains-bran ratios was studied. The fermentation substrate ratio was showed in Table 2.

**Table 2:** Fermentation substrate ratio.

Group	Distillers grains, %	Wheat bran, %	Corn, %	Soybean meal, %	Molasses, %
1	35	55	5	3	2
2	45	45	5	3	2
3	55	35	5	3	2
4	65	25	5	3	2
Control	0	90	5	3	2

#### 2.2.3 Optimization of fermentation conditions

In order to determine the material-to-liquid ratio, temperature and time, which were the most important elements in the fermentation, an orthogonal experiment with three factors and three levels was designed using Design-Expert8.0.6 software. Box–Behnken response surface optimization design was used, and the crude protein content after fermentation was used as response, response surface data fitting analysis was conducted to establish optimal fermentation conditions. The orthogonal test factor level table was showed in Table 3. The Box–Behnken response surface optimization design was showed in Table 4.

**Table 3:** Orthogonal test factor level table.

Level	(A) Moisture content, %	(B) Temperature, °C	(C) Time, day
1	1:1.1	28	5
2	1:1	32.5	7
3	1:0.9	37	9

**Table 4:** Box–Behnken response surface optimization design experiment.

Run	(A) Moisture content, %	(B) Temperature, °C	(C) Time, day
7	1:1.1	32.5	9
10	1:1.1	37.0	7
13	1:1	32.5	7
14	1:1.1	32.5	5
15	1:1	37.0	9
11	1:0.9	28.0	7
9	1:1	37.0	5
8	1:1	32.5	7
3	1:1	28.0	5
16	1:1.1	28.0	7
12	1:1	32.5	7
5	1:1	28.0	9
2	1:1	32.5	7
1	1:0.9	32.5	5
4	1:1	32.5	7
17	1:0.9	37.0	7
6	1:0.9	32.5	9

#### 2.2.4 Distillers grains fermentation experiment under optimized conditions

After a series of previous experiments, the optimal substrate ratio for probiotic-enzyme collaborative fermentation of distillers grains, as well as the fermentation material-to-liquid ratio, temperature and time with the best conversion effect was obtained. Fermentation was carried out again under the optimal fermentation conditions optimized above, and the crude protein, trichloroacetic acid soluble protein, crude fiber, acid detergent fiber, neutral detergent fiber, acid detergent lignin, fat and pH value of the system before and after fermentation were measured to evaluate the transforming effect of probiotic enzyme synergy on the nutrient composition of the system before and after fermentation. Contents of organic acids before and after fermentation, including lactic acid, acetic acid, propionic acid and butyric acid were determined. The anti-nutritional factors of mannan and xylan in the feed before and after fermentation were also determined. In addition, mycotoxins including aflatoxin B1, deoxynivalenol (DON) and zearalenone were measured before and after fermentation.

#### 2.2.5 Determination of nutrients

##### 2.2.5.1 Determination of crude protein and trichloroacetic acid soluble protein

According to GB/T 6432–2018, the Kjeldahl nitrogen determination method is used to determine crude protein and acid-soluble protein. First, the sample is digested at 400°C for 4 h in a digestion instrument. After cooling, it is placed in a KF1100 Kjeldahl nitrogen determination instrument for titration to obtain the nitrogen content. The protein content can be obtained by multiplying the nitrogen content by the conversion factor of 6.25. The crude protein sample is directly digested. Before measuring the acid-soluble protein, 5 g of the sample should be dissolved in 100 mL of 15% trichloroacetic acid solution, shaken at 150 rpm for 30 min, and left to stand for 5 min. The upper solution was centrifuged at 4,000 rpm for 5 min to obtain a supernatant sample and digest it.

##### 2.2.5.2 Determination of fiber content

The filter bag method was used to determine crude fiber, neutral detergent fiber (NAF), acid detergent fiber (NDF) and acid detergent lignin (ADL) according to GB/T 6434–2022. Place the sample in the F2000 fiber digestion instrument and add the corresponding digestion solution according to the different measurement indicators for digestion. Weigh the mass before and after digestion, and obtain the result based on the difference. According to the formula provided by National Renewable Energy Laboratory (NREL), the hemicellulose content and cellulose content of the sample is obtained: hemicellulose content = acid fiber content − neutral fiber content; cellulose content = acid fiber content − acid lignin content.

##### 2.2.5.3 Determination of fat content

The fat of the sample was determined to use Soxhlet extraction method according to GB/T 6433–2006. Place the sample in the SOX606 fat analyzer, use petroleum ether with a boiling range of 60–80°C for extraction, and extract at 90°C for 6 h. Dry the receiving cup to constant weight, and the fat content of the sample can be obtained from the mass difference before and after measurement.

#### 2.2.6 Determination of anti-nutritional factors

##### 2.2.6.1 Determination of mannan content

The phenol method was used to determine the mannan content. Dissolve the sample in 0.6% sodium hydroxide solution, place in a 75°C water bath for 30 min, cool and filter, and dilute to volume to obtain a sample solution. Take 1 mL of the sample solution and add 1.0 mL of 5% phenol solution. After mixing, add 5.0 mL of concentrated sulfuric acid, shake and mix, place in a boiling water bath for hydrolysis for 20 min, take out and cool, and measure the absorbance value at a wavelength of 490 nm. The standard curve operation is the same. Calculate the mannan content of the sample according to the linear regression equation of the standard curve.


$$\mathrm{Mannan}\;\mathrm{content}\;\left(\%\right)=\frac{C\times 10^{-3}\times 0.9\times 100}{M}$$

In the formula, C is the mannose content (μg/ml) of the sample calculated from the standard curve; M is the sample mass (g); 0.9 is the coefficient for converting mannose into mannan.

##### 2.2.6.2 Determination of arabinoxylan content

The arabinoxylan content was determined by the lichenol-hydrochloric acid method. Weigh about 100 mg of the sample into a 25 mL stoppered test tube, add 2 mL of 2 mol/L HCl, hydrolyze in a boiling water bath (100°C) for 2 h, filter into a 100 mL volumetric flask, and wash the residue with a small amount of distilled water several times (the washing liquid is also added volumetric flask) and dilute to volume (filter to volume). Take 1 mL of hydrolyzate and add 2.0 mL of distilled water, then add 3 mL of 0.1% FeC1_3_ and 0.3 mL of 1% lichenol, and react in a boiling water bath for 40 min. Take out tap water and cool for 10 min. Use a 1.0 cm cuvette to perform the test at 580 nm and 670 nm wavelengths. Measure the absorbance. For samples whose absorbance exceeds the standard curve, dilute the hydrolyzate to within the range of the standard curve.


$$\mathrm{Xylan}\;\mathrm{content}\;\left(\%\right)=\frac{\left[\left(A-b\right)/a\right]\times n\times 0.88\times 100}{m}$$

In the formula, a and b constants (obtained from the regression equation of the standard curve); A is the absorbance difference of the sample at 670 and 580 nm; 0.88 is the polymerization coefficient; n is the dilution factor; m represents the mass.

#### 2.2.7 Determination of organic acids

The determination of organic acids was based on the method of Scherer with slight modifications (Scherer et al., 2012). Weigh 5 g of the sample and add 35 mL of distilled water, shake in a constant temperature shaking incubator (200 rpm, 4°C), and centrifuge for 15 min (4°C, 10,000 g) after 24 h. The collected supernatant was filtered using a 0.22 micron filter membrane and determined by HPLC (Agilent). The chromatographic column is an Agilent C_18_ column (250 mm × 4.6 mm, 5 μm), the mobile phase A is methanol, and the mobile phase B is 0.02 mol/L NaH_2_PO_4_ (phosphoric acid adjusts the pH to 2.70). Isogradient elution, the mobile phase is 14% A, 86% B, the running time of a single sample is 25 min, the flow rate is 1 mL/min, the column temperature is 30°C, the detection wavelength is 210 nm, and the injection volume is 20 μL. Standard lactic acid, acetic acid, propionic acid, and butyric acid are all chromatographically pure (sigma).

#### 2.2.8 Determination of mycotoxins

The mycotoxins detected in this experiment: aflatoxin B1, zearalenone, and DON were all measured using PriboFast^®^ ELISA detection kits, which were purchased from Qingdao Pribolab Company.

#### 2.2.9 Data analysis

The data were analyzed by a one-way analysis of variance using the General Linear Models in SPSS20.0 software. Design-Expert8.0.6 software was used to design the response surface optimization experiment and analysis data fitting out the best fermentation conditions. *p*-value of 0.05 was used to indicate of a significant difference. The results are expressed as the means and standard deviations.

## 3 Results

### 3.1 Optimization of fermentation substrate ratio

As shown in Table 5, the effect of water content on fermentation results was explored. When the moisture content was 50% (group 3), the crude protein content increased by 28.7% and the acid-soluble protein content increased by 504%. At this time, the crude protein and acid-soluble protein of the fermented sample increased most significantly.

**Table 5:** The influence of different moisture content on fermented traditional feed.

Group (moisture)	CP, %			TCA-SP, %		
	Raw feed	Fermented feed	Change rate	Raw feed	fermented feed	Change rate
1 (10%)	23.78	23.70	−0.3%	2.03	1.74	−14.3%
2 (30%)	23.78	29.88	25.6%	2.03	6.76	233%
3 (50%)	23.78	30.62	28.7%	2.03	12.27	504%

As shown in Table 6, the impact of different distillers grains-bran ratios on the fermentation effect was explored. The results showed that the crude protein of group 2 increased by 15.8% after fermentation, and the crude protein improvement effect was the best. The acid-soluble protein in group 1 increased by 75.9% compared with that before fermentation, and the acid-soluble protein in experimental group 2 increased by 68.3% compared with that before fermentation. Group 1 and 2 performed excellently. The ratio of experimental group 2 (45% distillers grains, 45% wheat bran, 5% corn, 3% soybean meal, and 2% molasses) was selected as the substrate ratio for subsequent experiments.

**Table 6:** The influence of different distillers grains-bran ratios on fermented traditional feed.

Group	CP, %			TCA-SP, %		
	Raw feed	fermented feed	Change rate	Raw feed	fermented feed	Change rate
1	21.37	22.77	6.6%	3.11	5.47	75.9%
2	21.44	24.82	15.8%	3.29	5.54	68.3%
3	21.51	22.35	3.9%	3.47	5.70	64.3%
4	21.57	24.50	13.6%	3.65	5.35	46.6%
Control	21.14	22.28	5.4%	2.46	5.53	124.8%

### 3.2 Fermentation condition response surface optimization results

CP content of 17 fermented groups designed by Box–Behnken response surface optimization were showed in Table 7. The CP content of Run 13 is the highest in the 17 fermented groups, 17.06%.

**Table 7:** Response surface optimization design experimental results.

Run	(A) Moisture content, %	(B) Temperature, °C	(C) Time, day	CP, %
7	1:1.1	32.5	9	16.78
10	1:1.1	37.0	7	16.37
13	1:1	32.5	7	17.06
14	1:1.1	32.5	5	16.99
15	1:1	37.0	9	16.38
11	1:0.9	28.0	7	16.95
9	1:1	37.0	5	16.41
8	1:1	32.5	7	16.95
3	1:1	28.0	5	16.51
16	1:1.1	28.0	7	17.02
12	1:1	32.5	7	16.12
5	1:1	28.0	9	16.21
2	1:1	32.5	7	16.42
1	1:0.9	32.5	5	16.61
4	1:1	32.5	7	16.73
17	1:0.9	37.0	7	16.66
6	1:0.9	32.5	9	16.81

**Figure 1:**
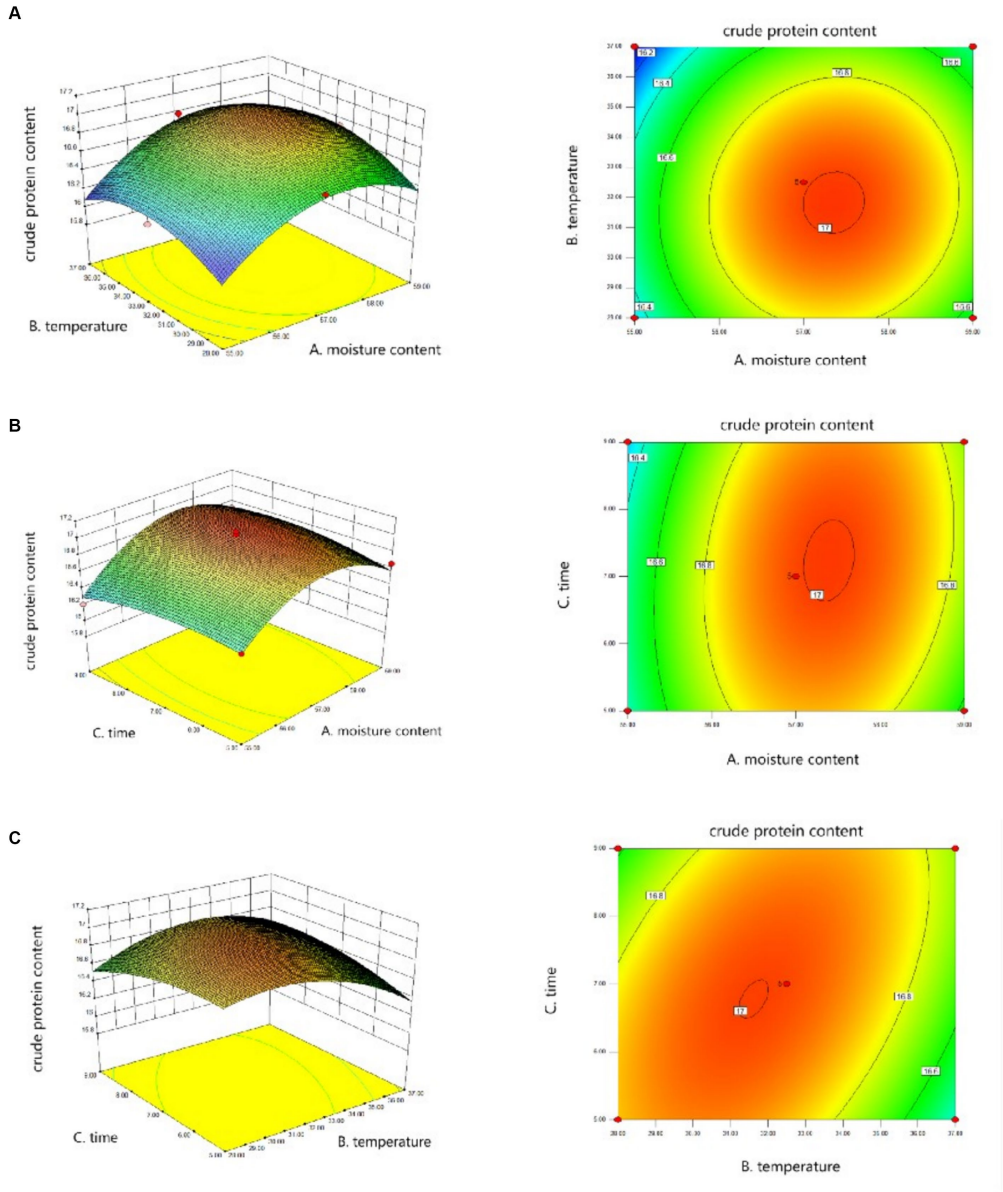
Response surface data fitting diagram. **(A)** Surface and contour plots of the interaction effect of moisture content and temperature on crude protein content. **(B)** Surface and contour plots of the interaction effect of moisture content and time on crude protein content. **(C)** Surface and contour plots of the interaction effect of temperature and time on crude protein content.

Figure 1 showed the interaction between different variables, and Design-Expert software gave the fitting formula Y = −298.17703 + 10.91006 × A + 0.40698 × B − 1.18813 × C + 0.00444444 × AB +0.016875 × AC + 0.017500 × BC − 0.097375 × A^2^ − 0.012321 × B^2^ − 0.024250 × C^2^, A is the moisture content, B is the temperature, and C is the fermentation time. Among them, the relationship between temperature and material-liquid ratio, time and material-liquid ratio is significant, but the relationship between temperature and time is not obvious. According to the Design-Expert software prediction, the fermentation effect is best when the moisture content of the fermentation system is 57.35%, the temperature is 31.81°C, and the time is 6.98 days. The predicted value of crude protein content at this time is 17.01%, and the credibility is 95%.

### 3.3 Amplify experimental results

Based on the optimal fermentation results obtained from response surface analysis, some modifications were made to comply with actual production conditions. It was finally determined that the water content was 57% (the material-to-liquid ratio was 1:1), 31.8°C, and 7 days as the conditions for scale-up production. And a series of indicators of the distillers grains after the amplification experiment were tested to determine whether the feeding value of the fermented distillers grains has been improved.

### 3.4 Nutrient analysis

As shown in Table 8, the crude protein content of distillers grains after fermentation significantly increased from 15.55 to 20.41%, an increase of 31.25%, and the acid-soluble protein content increased from 1.99 to 2.72%, an increase of 36.68%. The fat content of distillers grains after fermentation is 4.19%, which is 49% higher than 2.81% before fermentation. The difference is extremely significant. In this study, the *CF*, NAF, NDF, and ADL contents before fermentation were 24.65, 59.82, 34.10, and 12.46%, respectively. The cellulose content and hemicellulose content before fermentation were 21.64 and 25.72%, respectively. These complex polysaccharides can be degraded into simple sugars by enzymes and microorganisms. After fermentation, the crude fiber content of distillers grains was 43.72%, a significant decrease of 34%. After fermentation, the NAF, NDF, and ADL contents were 43.72, 22.78, and 8.68% respectively, all of which showed significant decreases, with respective decreases of 26.91, 33.20, and 30.34%. The converted cellulose content was 14.1%, a decrease of 34.84%; the hemicellulose content after fermentation was 20.98%, a decrease of 16.41%. The pH of the lees dropped from 6.16 before fermentation to 4.23, and the difference was extremely significant.

**Table 8:** Effect of fermentation on the concentration of nutrients.

Item, %	Group			*P*-value
	Raw mixed feed	Fermented feed	Change rate	
CP	15.55 ± 0.11	20.41 ± 0.16	31.25%	<0.001
TCA-SP	1.99 ± 0.13	2.72 ± 0.27	36.68%	0.039
Fat	2.81 ± 0.27	4.19 ± 0.38	49.11%	<0.001
CF	24.65 ± 0.15	16.21 ± 0.26	−34.24%	0.007
NAF	59.82 ± 0.79	43.72 ± 2.08	−26.91%	0.003
NDF	34.10 ± 2.55	22.78 ± 0.15	−33.20%	<0.001
ADL	12.46 ± 1.01	8.68 ± 0.74	−30.34%	<0.001
pH	6.16 ± 0.17	4.23 ± 0.05	−31.33%	<0.001

### 3.5 Analysis of anti-nutritional factors

The analysis of anti-nutritional factors was showed in Table 9, the mannan content decreased from 12.09 to 8.83% after fermentation, a decrease of 26.96% that indicated the difference was extremely significant, and the arabinoxylan content decreased from 4.82 to 2.85% after fermentation, a decrease of 40.87% that indicated the difference is significant.

**Table 9:** Effect of fermentation on the concentration of anti-nutritional factors.

Antinutritional factors, μg/mL	Group			*P*-value
	Raw mixed feed	Fermented feed	Change rate	
Mannan	12.09 ± 0.89	8.83 ± 0.55	−26.96%	<0.001
Arabinoxylan	4.82 ± 1.34	2.85 ± 1.03	−40.87%	0.025

### 3.6 Analysis of organic acids

**Figure 2:**
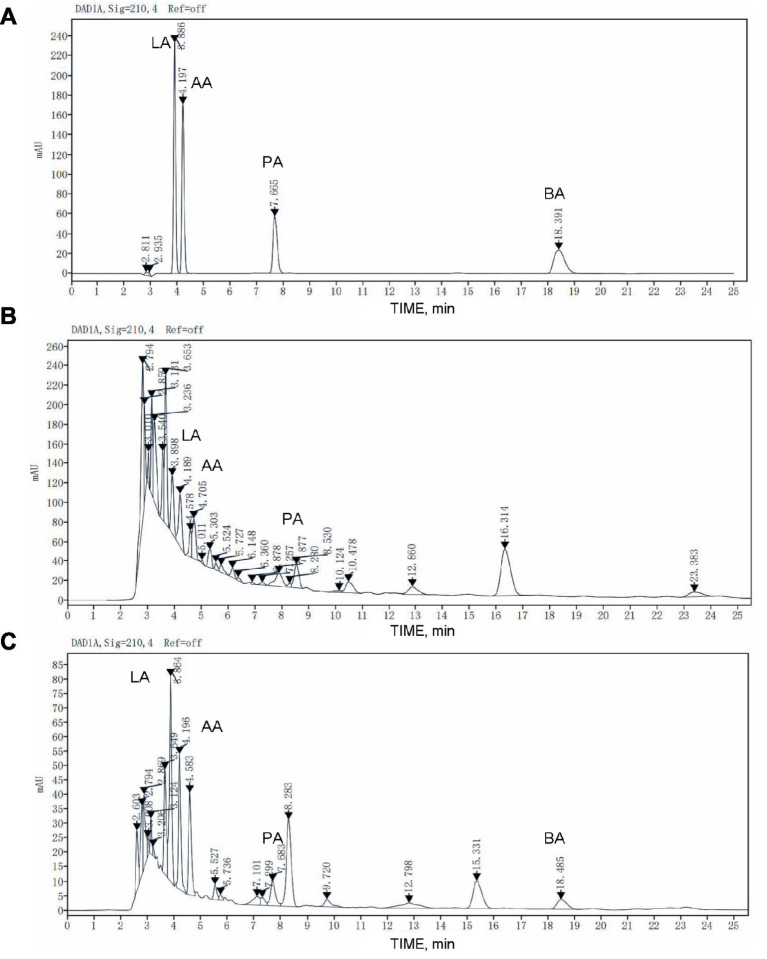
HPLC chromatogram of organic acid in the raw feed and fermented feed. **(A)** The chromatogram of organic acid standard substance. **(B)** The raw feed. **(C)** The fermented feed. LA, lactic acid; AA, acetic acid; PA, propionic acid; BA, butyric acid.

As shown in Figure 2, The retention time of lactic acid is 3.886 min, that of acetic acid is 4.197 min, that of propionic acid is 7.665 min, and that of butyric acid is 18.391 min. Due to the complex composition of the sample, it is difficult to separate each peak shape and determine the retention time of the organic acid in feed samples completely. The organic acid peak elution time of samples before and after fermentation can be determined by adding a standard mixture of known concentration to the sample as an internal standard.

As shown in Table 10, the organic acid (lactic acid, acetic acid, propionic acid and butyric acid) contents of the samples after fermentation increased significantly. The lactic acid content increased from 2.59 mg/g before fermentation to 8.83 mg/g, an increase of 240.93%. The acetic acid content increased from 3.10 mg/g before fermentation to 5.48 mg/g, an increase of 76.77%. The content of propionic acid after fermentation was 1.80 mg/g, which was 89.47% higher than the 0.95 mg/g before fermentation. Butyric acid was not detected before fermentation, and the butyric acid content after fermentation was 0.51 mg/g.

**Table 10:** Effect of fermentation on the concentration of organic acids.

Organic acid, mg/g	Group			*P*-value
	Raw mixed feed	Fermented feed	Change rate	
Lactic	2.59 ± 0.01	8.83 ± 0.03	240.93%	<0.001
Acetic	3.10 ± 0.03	5.48 ± 0.01	76.77%	<0.001
Propionic	0.95 ± 0.01	1.80 ± 0.01	89.47%	<0.001
Butyric	ND	0.51 ± 0.01		<0.001

### 3.7 Mycotoxin analysis

As shown in Table 11, the contents of DON and Aflatoxin B1 in distillers grains before and after fermentation have almost no change, but the DON and Aflatoxin B1 in feed after fermentation are both within the scope of the national feed safety standards, and the national standards are 5,000 and 30 μg/kg, respectively. The zearalenone content after fermentation decreased from 8.70 to 6.80 μg/kg compared with before fermentation. Although the difference was not significant, it showed a decreasing trend, the content of mycotoxin before and after fermentation was within the national safety standard of 1,000 μg/kg (Gong et al., 2017).

**Table 11:** Effect of fermentation on the concentration of mycotoxins.

Mycotoxin, μg/kg	Group			*P*-value
	Raw mixed feed	Fermented feed	The limit standard	
DON	998.27 ± 18.69	1007.87 ± 14.20	<5,000	0.34
Aflatoxin B1	10.53 ± 2.69	10.63 ± 0.97	<30	0.944
Zearalenone	8.70 ± 2.15	6.80 ± 2.77	<1,000	0.405

## 4 Discussion

During the fermentation process, microorganisms are very sensitive to temperature and moisture. Normally, *Aspergillus oryzae* and *Saccharomyces cerevisiae* maintain vigorous vitality at 28–30°C, while the optimal growth temperature of *Lactobacillus casei* and *Bacillus subtilis* is generally 37°C (Alonso, 2016). The four probiotic strains used in this study have different suitable growth environments. When the temperature is 28 and 37°C, the response of fermentation is not as good as that at 32.5°C. At 32.5°C, all the four strains grew under relatively mild conditions. Although this strains were not grown under their respective optimal growth temperatures, they showed the ability to degrade polysaccharides such as cellulose and convert them into proteins through metabolism. The material-to-liquid ratio, that is, the moisture content, affects the fermentation significantly. High moisture reduces the porosity of the matrix, promotes particle aggregation and inhibits gas exchange. Conversely, low moisture can negatively impact microbial metabolism by reducing the stability of extracellular enzymes and limiting nutrient solubility (Ezeilo et al., 2019). Although the correlation between fermentation time and temperature is not significant, it is also an important factor affecting the fermentation results. The fermentation time of 7 days is better than that of 5 and 9 days. This maybe because the growth rate of the mold was slow resulting the growth of *Aspergillus oryzae* had not reached its optimal state at 5 days (Zuo et al., 2023). The insufficient biomass leads to insufficient protein conversion ability. At 9 days, it may be because the fermentation time is too long, resulting in too much biomass, which competes for substrates to maintain the growth and development of the probiotic, and part of the transformed nutrients are consumed and wasted by the probiotic. Liu optimized a better distiller’s grain fermentation process through single factor and orthogonal experiments, and found that the fermented distiller’s grain produced under these conditions has better feeding value (Liu et al., 2023). Therefore, the above-mentioned suitable fermentation conditions are conducive to the production of high-quality and stable fermented feed.

Ibarruri reported that the natural protein in distillers grains increased by 18–24% through SSF in 7 days (Ibarruri et al., 2019), lower than in this research, indicating that the probiotic-enzyme collaborative fermentation technology used in this study had good ability to improve the nutritional value of distillers grains. During the fermentation process, microorganisms may degrade polysaccharides in distillers grains into monosaccharides or oligosaccharides, Therefore, DDGS often contain higher concentrations of non-fermentable components such as fats and proteins, which is consistent with the previous report of Rho et al. (2020). Liu et al. (2023) reported a study on the use of microbial flora to degrade crude fiber of distillers grains. The results showed that the conversion rates of cellulose and hemicellulose were 19.64 and 10.88%, respectively. Astudillo-Neira et al. (2022) reported that carbohydrates from lignocellulosic feed can be released by fungi for fermentation. Datsomor et al. (2022) reported that the *P. ostreatus* treated straw had a lower lignin content (3.3%) compared to the *P. chrysosporium* (6.2%) with the raw straw recording the 8.2% lignin fraction. Wei et al. (2023) reported that under the optimal parameters for fungal fermentation of *Codonopsis pilosula* straw, the cellulose degradation rate and lignin degradation rate reached 13.65 and 10.73%, respectively. These reports are consistent with our research results, proving that cellulose, hemicellulose and lignin in distillers grains can be degraded by microorganisms into simple sugars such as glucose and xylose. The pH after fermentation is significantly lower than before fermentation. A lower pH value can inhibit the growth of miscellaneous probiotic and reduce the adverse effects on the fermentation effect. At the same time, a lower pH value can improve the palatability of the feed (Nguyen et al., 2020). This may be due to the fact that *Lactobacillus casei* fermentation produces a large amount of lactic acid during the fermentation process, which plays a crucial role in quickly lowering the pH, and acetic acid, propionic acid and butyric acid are also produced after fermentation, which can all lower the pH.

The reason why the anti-nutritional factors showed a significant decrease was the addition of 0.1% complex enzyme, which included mannanase and arabinoxylanase, which had a directional degradation effect on these two anti-nutritional factors (Zangaro et al., 2019). However, the effect of adding complex enzymes exclusively was not significant generally due to the solid-state fermentation. According to the research of Kapoor et al. (2019), the efficiency of enzymatic hydrolysis increases with the increase of enzyme loading and decreases with the increase of solid loading. It is speculated that among the four strains of probiotics added in this experiment, some strains had the ability to degrade polysaccharides such as mannan and arabinoxylan. This also shows that the synergistic effect of probiotic and enzymes on improving the properties of distillers grains is better than that of adding enzyme preparations only.

The lactic to acetic ratio (the ratio of lactic acid content to acetic acid content) is an important indicator for evaluating the fermentation effect of feed. It is generally believed that the fermentation is abnormal when the ratio is less than 1 (Canibe et al., 2010). The ratio of lactic to acetic in this experiment increased from 0.83 to 1.61 after fermentation. *Lactobacillus casei* fermentation produces a large amount of lactic acid, which plays a vital role in lowering pH rapidly, thereby inhibiting the growth of aerobic harmful probiotic and mold and reducing nutrient loss. The increase in acetic acid content during the fermentation process is beneficial to improving the aerobic stability of the fermentation process and inhibiting the reproduction of some undesirable probiotic (Kim and Lee, 2021). We believed that the fermented distillers grains at this time have a lower pH value, which can inhibit the contamination of miscellaneous probiotic, and the lactic acid has a special fragrance and acidic taste, which is beneficial to enhancing the feed’s palatability and animals’ willingness to ingestion (Yang et al., 2006). Propionic acid is one of the metabolites of pig intestinal microorganisms. It plays an important role in regulating body health and metabolism, such as anti-inflammatory factors. It can enhance intestinal health and function, growth performance and overall health in many aspects, and is often used in the mildew-proof (Longpré et al., 2016). Butyric acid plays an energy supply role in the hind intestine of pigs, and can also significantly reduce the pH value of the intestine, significantly reduce the number of harmful probiotic such as *Escherichia coli* and *Salmonella*, and improve the diarrhea problem of weaned piglets. However, butyric acid is easily oxidized and denatured, producing a pungent odor. A trace amount of butyric acid was produced after fermentation in this experiment, which had no significant impact on the aromatic smell of the fermented feed (Słupecka-Ziemilska et al., 2022; Uddin et al., 2023). Biagi et al. (2007) reported that adding a small amount of butyric acid as a feed additive to the feed can improve the microbial intestinal flora of piglets and immune system.

There was almost no change in the contents of DON and Aflatoxin B1 in the distillers grains before and after fermentation, indicating that the strain may lack the ability to degrade the above two toxins or the toxin content is too low, resulting in insignificant fermentation degradation effect. The content of zearalenone showed a decreasing trend after fermentation. In short, although fermentation has no significant effect on reducing mycotoxins, the mycotoxin indicators after fermentation are within the range of national standards (DON<5,000 μg/kg, Aflatoxin B1 < 30 μg/kg, zearalenone<1,000 μg/kg), so fermented distillers grains can be considered safe and harmless as feed (Binder et al., 2007).

## 5 Conclusion

Distillers grains are an agricultural and sideline product with high feeding potential. The synergistic solid-state fermentation technology of probiotic enzymes can effectively improve the feeding performance of distillers grains. Under the fermentation conditions of 57% moisture content, 31.8°C, and 7 days, the fermented mixed feed showed the highest crude protein content, the anti-nutritional factors were reduced significantly, the organic acid content was increased significantly, and the performance as a feed material was greatly improved. This study provides a potential way for the high-value development of distillers grains.

## Data availability statement

The raw data supporting the conclusions of this article will be made available by the authors, without undue reservation.

## Author contributions

KC: Data curation, Investigation, Writing – original draft. XD: Writing – review & editing, Investigation, Methodology. DJ: Writing – review & editing, Validation. LQ: Writing – review & editing, Investigation. ML: Writing – review & editing, Investigation. MY: Writing – review & editing, Software. WJ: Writing – review & editing, Software. LZ: Writing – review & editing, Project administration. JJ: Conceptualization, Writing – review & editing, Supervision. LL: Supervision, Writing – review & editing, Project administration.
